# Identifying subtypes and determinants of fall risk perception in older adults: a latent profile analysis

**DOI:** 10.3389/fpubh.2026.1759157

**Published:** 2026-03-26

**Authors:** Xin Yang, Haiyan Xiang, Weiming Qian, Qin Xu, Yuping Zhang, Haotian Chen, Xiaoxu Han, Meiqi Yao

**Affiliations:** Department of Nursing, The Second Affiliated Hospital of Zhejiang University School of Medicine, Hangzhou, China

**Keywords:** fall risk, fall, healthy aging, older adults, risk perception

## Abstract

**Background:**

Falls have long been a significant safety concern worldwide, not only compromising the physical and psychological health of older adults and limiting their social engagement but also imposing substantial economic and caregiving burdens. Evidence on fall risk perception among Chinese community-dwelling older adults remains limited, especially for those transitioning to community living after hospital discharge.

**Objective:**

This research examined the subtypes of fall risk perception of Chinese community-dwelling older adults in the post-discharge transition and to explore subgroup characteristics and associated factors.

**Methods:**

A cross-sectional survey was conducted between January 2024 to March 2025 in Hangzhou, Zhejiang Province. A self-designed questionnaire was used to collect demographic and health-related information, The Fall Risk Perception Scale for Community-dwelling Older Adults was used to assess the fall risk perception, the objective fall risk was assessed by Morse Fall Scale. Latent profile analysis (LPA) was performed to extract latent classes of fall risk perception, and multinomial regression analyses were used to identify differences between these categories.

**Results:**

A total of 468 older adults were included, with 56.0% were male. Three fall risk perception subtypes were identified by LPA: Low Perception-Social Context Desensitized Type (29.2%), Moderate Perception - Balanced Type (43.4%), and High Perception - Bio-behaviorally Salient Type (27.4%). Individuals who were aged with 70–79 (OR = 0.46, 95% CI: 0.27–0.77), with college education or above (OR = 0.31, 95% CI: 0.13–0.76), those who underwent surgery during hospitalization (OR = 0.26, 95% CI: 0.15–0.43), reported difficulty falling asleep (OR = 0.40, 95% CI: 0.20–0.82), and those with a history of falls (OR = 0.44, 95% CI: 0.24–0.81) were significantly more likely to be in the High Perception - Bio-behaviorally Salient Type. Compared to objective fall risk level, a third of participants (31.4%) correctly estimated their fall risk, 23.1% overestimated it and 45.5% underestimated it.

**Conclusion:**

Most older adults possess a Moderate Perception - Balanced Type toward fall risk. Key determinants of heightened risk perception included advanced age, higher education, fall history, and recent surgical experience. Tailored, profile-specific risk communication strategies are essential to improve perceptual accuracy during the hospital-to-home transition may support post-discharge fall prevention.

## 1 Background

According to the World Health Organization (WHO), the number of people aged 60 years and older is expected to double by 2050, reaching over 2 billion worldwide (1). This demographic shift is progressing faster than ever before and is projected to accelerate even further in the coming decades, especially in low- and middle-income countries (1). This trend poses significant public health challenges, among which falls have emerged as a leading cause of morbidity and mortality in older adults. Aging is associated with various physiological changes, including declines in muscle strength, balance, and cognitive function, all of which contribute to an elevated risk of falls by 7% annually (2).

It is estimated that approximately one-third of community-dwelling older adults experience at least one fall each year (0.67 falls per person per year), with around 10% experiencing multiple falls annually (3). More than 10% of falls lead to injuries—including fractures, dislocations, head trauma, and functional decline (4, 5), and are associated with substantial economic burden and significant social burden, with annual medical costs estimated at $49.5 billion (6). In addition, falls are associated with various psychological consequences among older adults, such as fear of falling, depression, and social withdrawal, which in turn contribute to a decline in their overall quality of life (7). Importantly, many of these falls are preventable with appropriate risk assessment and targeted interventions (8). This need may be especially salient during the hospital-to-home transition after discharge, when older adults return to community living and re-establish fall-prevention self-management.

Although numerous fall prevention programs have achieved measurable success by targeting objectively assessed fall risk using tools (e.g., Morse Fall Scale) (9), increasing attention is being paid to patients’ subjective perception of fall risk, a process in which individuals estimate their chances of falling and then decide whether the risk is significant enough to take seriously (10). Among older adults, this perception is particularly critical, as it can directly influence adherence to safety instructions, engagement in physical activity, and willingness to participate in fall prevention programs (10–12). However, older adults often misunderstand their fall risk—either underestimating or overestimating it—due to cognitive limitations, impaired insight, or psychological factors such as denial or anxiety (13–15). Recent studies have highlighted a considerable gap between clinical risk assessments and patients’ self-perceived fall risk. For instance, Kuhlenschmidt et al. found that about one-third of hospitalized cancer patients underestimated their fall risk, perceiving themselves to be at low risk despite being rated as high risk by nurses (16). Similarly, in a study of 253 patients identified as high-risk, only 63.2% were aware of their own higher vulnerability to falls (14). This lack of risk perception has been associated with increased fall incidence and decreased adherence to fall prevention strategies, underscoring the need to explore the cognitive and behavioral underpinnings of fall risk misperception in older adults (17).

From a theoretical perspective, fall risk perception can be viewed as an individual’s appraisal of susceptibility to falling and the anticipated severity of its consequences, formed through the integration of bodily cues (e.g., dizziness, gait instability), prior experiences (e.g., falls), and contextual information (e.g., home environment) (10, 14, 18). Classic health-behavior frameworks (e.g., the Health Belief Model and Protection Motivation Theory) posit that such risk appraisals shape preventive intentions and actions by influencing perceived threat and coping motivation, thereby affecting engagement in safety behaviors and self-management (19, 20). Importantly, risk perception is not purely a reflection of objective risk; it may be biased by cognitive limitations, affective responses (e.g., fear, anxiety), and motivational processes (e.g., optimistic bias or denial), which helps explain why some older adults underestimate risk while others overestimate it (21). Therefore, conceptualizing fall risk perception as a multi-component, potentially heterogeneous construct provides a theoretical rationale for adopting a person-centered approach to identify distinct perception patterns and their behavioral implications.

Despite its clinical relevance, fall risk perception is often treated as a unidimensional variable in existing studies (14, 15, 22), overlooking the potential heterogeneity in how older patients perceive their risk. Some individuals may display an optimistic bias, minimizing their vulnerability, while others may experience excessive fear or hypervigilance. A person-centered analytic approach, such as Latent Profile Analysis (LPA) (23), can reveal distinct subgroups based on risk perception patterns, allowing for a more nuanced understanding of patients’ needs and facilitating personalized interventions (21). Furthermore, limited research has explored the factors associated with different risk perception profiles, such as demographic characteristics, physical function, history of falls, and social support. Identifying these determinants is particularly important during the hospital-to-home transition, when older adults return to community living and must translate risk appraisal into effective fall-prevention self-management; this may help clinicians identify individuals prone to risk-perception bias and tailor communication or behavioral interventions accordingly (10).

Therefore, this study aimed to (1) identify potential subtypes of fall risk perception among older adults transitioning from hospital to community living after discharge using LPA, and (2) examine the sociodemographic, clinical, and psychosocial factors associated with these subtypes. The findings are expected to inform the development of profile-informed fall-prevention strategies and to strengthen risk communication and self-management support during the hospital-to-home transition.

## 2 Materials and methods

### 2.1 Study design and participants

**Figure 1:**
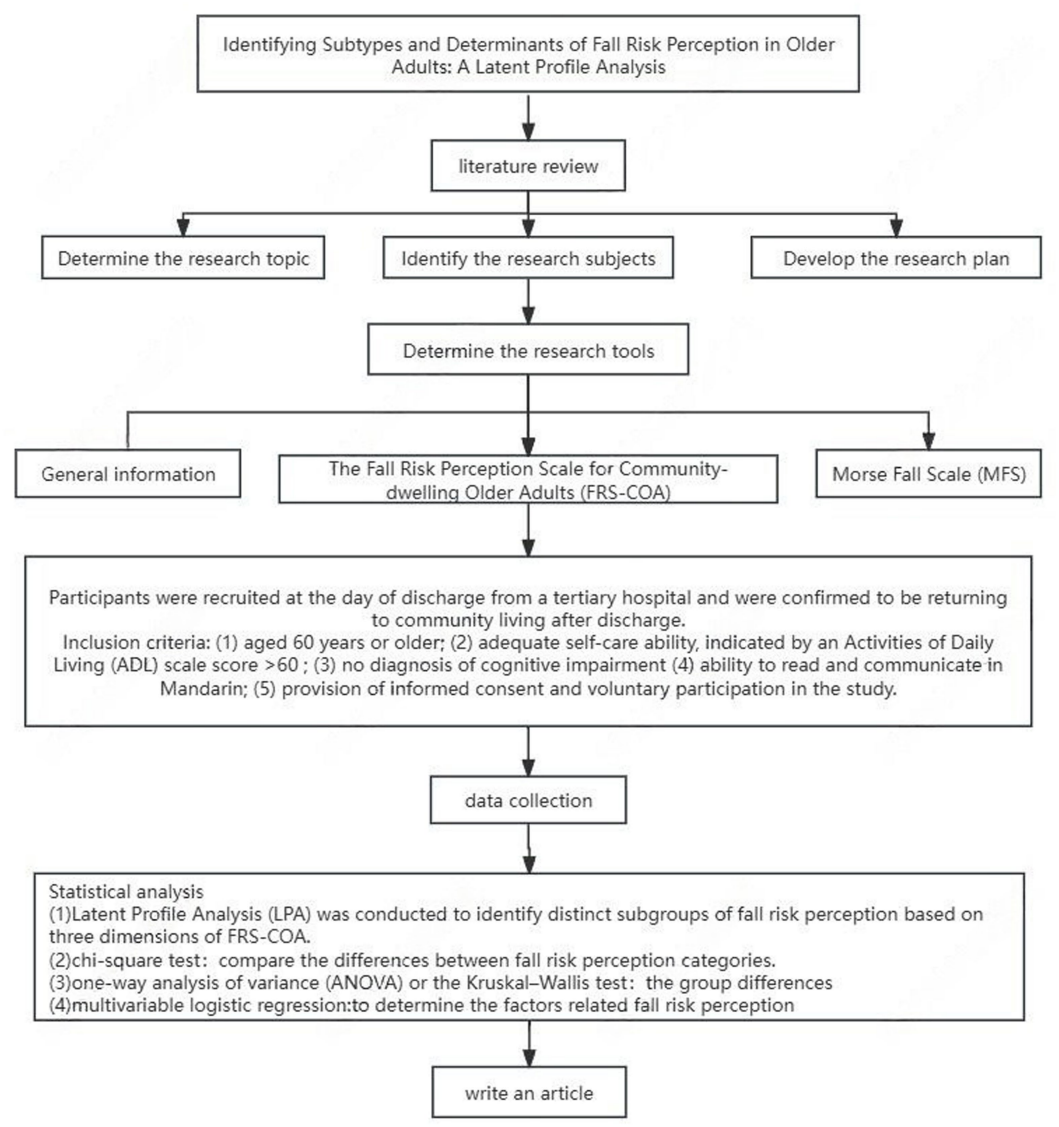
Research flowchart.

A cross-sectional survey was conducted between January 2024 to March 2025 in Hangzhou, Zhejiang Province. Participants were recruited at the day of discharge from a tertiary hospital and were confirmed to be returning to community living after discharge. The research flowchart was shown in Figure 1. The inclusion criteria were: (1) aged 60 years or older; (2) adequate self-care ability, indicated by an Activities of Daily Living (ADL) scale score >60 (24); (3) no diagnosis of cognitive impairment (e.g., Alzheimer’s disease, post-stroke cognitive impairment, and dementia, et al.); (4) ability to read and communicate in Mandarin; (5) provision of informed consent and voluntary participation in the study. Participants were excluded if they had severe vestibular dysfunction, major visual impairment, or other conditions affecting gait or balance; had severe or unstable medical conditions that precluded participation; or had a history of substance abuse.

Sample size: According to Kendall’s estimation method, the required sample size was calculated as 5 to 10 times the number of variables included in the analysis (25). Given that 21 variables were examined, the estimated sample size ranged from 105 to 210. To account for potentially invalid responses, a 20% buffer was added, resulting in a final estimated sample size requirement of approximately 126 to 252 participants. In addition, prior methodological work has suggested that LPA often benefits from larger samples (approximately 500) to support stable class enumeration and parameter estimation (26). After excluding questionnaires with incomplete or invalid responses, 468 participants were retained for the final analysis.

### 2.2 Data collection procedure

To capture fall risk perception during the hospital-to-home transition, participants were recruited on the day of hospital discharge. At this time point, individuals were preparing to resume community living after inpatient care, which is consistent with our approach of prioritizing early, upstream fall prevention at the point of community re-entry. This approach also facilitated timely recruitment and standardized data collection.

All data in this study were collected using paper-based questionnaires. Prior to data collection, the research team collaborated with the head nurses of each department to designate one nurse per unit as the survey coordinator. These coordinators were responsible for notifying the research staff when eligible patients were being discharged, thereby facilitating timely participant recruitment and data collection. They received standardized training to ensure a clear understanding of the study’s objectives, procedures, and data quality requirements. Data collection was conducted by trained research staff on the day of discharge, following a standardized protocol and strict quality control procedures. To ensure the completeness and reliability of the data, all questionnaire items were required to be fully completed prior to submission. The research was approved by the Ethics Committee of the Second Affiliated Hospital of Zhejiang University School of Medicine (No. 2023–0085). Informed consent was obtained from all participants, and the purpose and content of the study were clearly explained prior to questionnaire administration.

### 2.3 Measures

#### 2.3.1 General information

A self-designed questionnaire was used to collect demographic and health-related information, including gender, age, marital status, place of residence, monthly income, educational level, disease history (hypertension, heart disease, diabetes, or cerebrovascular disease), body mass index (BMI), smoking and drinking status, difficulty in falling asleep, mobility status, type of medical payment, living status, length of hospital stay, surgical procedures during hospitalization, and fall history prior to admission.

#### 2.3.2 The fall risk perception scale for community-dwelling older adults

The FRS-COA was originally developed by Bao et al., was used to assess the community-dwelling older adults’ self-perceived risk of falling (27). The questionnaire comprises 17 items across three dimensions: perceived fall-related biological behavior susceptibility (items 1–8), perceived fall-related severity consequences (items 9–13), and perceived fall-related social and environmental susceptibility (items 14–17). Responses are rated on a 5-point Likert scale ranging from 1 (“very unconcerned”) to 5 (“very concerned”), with total scores ranging from 17 to 85. Higher scores reflect a higher level of fall risk perception. The FRS-COA demonstrated strong psychometric properties, with a Cronbach’s *α* of 0.913 and subscale coefficients ranging from 0.814 to 0.858. The content validity index (CVI) was 0.940 (27). In the current study, the tool showed excellent internal consistency, with a Cronbach’s α of 0.964.

#### 2.3.3 Morse fall scale

The MFS developed by Professor Janice Morse in 1989, is a widely used, observer-rated instrument for assessing the risk of falls in hospitalized patients (28). The scale includes six items: a history of falling within the past 3 months, the presence of more than one medical diagnosis, use of ambulatory aids, intravenous therapy or administration of high-risk medications, gait characteristics, and mental status. Each item is assigned a weighted score, resulting in a total score ranging from 0 to 125. Fall risk is classified into three levels: low (0–24), moderate (25–44), and high (≥45) (29). The MFS has been extensively validated in clinical settings, demonstrating good reliability and validity, with inter-rater reliability ranging from 0.72 to 0.93, a Cronbach’s α of 0.891, sensitivity of 0.78, and specificity of 0.83 (28). Given that participants were evaluated on the day of discharge, the MFS was considered appropriate for identifying individuals at elevated fall risk prior to their return to the community. In this study, the scale was administered by ward nurses on the day of discharge and retrieved from the medical information system.

### 2.4 Statistical analysis

Latent Profile Analysis (LPA) was conducted to identify distinct subgroups of fall risk perception based on three dimensions of FRS-COA. The analysis was performed using the mclust (30) and tidyLPA packages (31) in R. LPA assumes the existence of an unobserved latent structure that classifies individuals into mutually exclusive and exhaustive categories (32). Model fit and the optimal number of latent classes were determined using multiple indices. These included: (1) Akaike Information Criterion (AIC), Bayesian Information Criterion (BIC), and sample size-adjusted BIC (aBIC), with lower values indicating better model fit (21); (2) Entropy, where values closer to 1 suggest greater classification precision (33); (3) Lo–Mendell–Rubin Likelihood Ratio Test (LMR-LRT) and Bootstrap Likelihood Ratio Test (BLRT), which compare models with k and k − 1 classes. A significant *p*-value (*p* < 0.05) indicates that the model with k classes fits the data significantly better (34).

All statistical analyses were performed using SPSS version 25.0. Categorical variables were presented as frequencies and percentages. We used chi-square test to compare the differences between fall risk perception categories. Continuous variables were summarized as a mean and standard deviations (SDs) for normally distributed data, or as medians with interquartile ranges for non-normally distributed data; and the group differences were assessed using one-way analysis of variance (ANOVA) or the Kruskal–Wallis test. To determine the factors related fall risk perception, multivariable logistic regression was employed. Independent variables were screened using univariable analyses, and only variables with *p* < 0.05 were entered into the multivariable model. Multicollinearity was assessed using VIF and tolerance (VIF > 5 or tolerance < 0.20); when present, redundant variables were removed. Spearman rank correlation analysis was conducted to examine the association between latent classes of fall risk perception and objective fall risk level. Correlation coefficients (|r|) were interpreted as follows: 0.10–0.30 as low, 0.31–0.60 as moderate, and 0.61–1.00 as high (35). A two-sided *p*-value < 0.05 was considered statistically significant.

## 3 Results

### 3.1 Sample characteristics

Table 1 presents the demographic and clinical characteristics of the enrolled older adults. Among the 468 participants, 262 (56.0%) were male, and 272 (58.1%) were aged 60–69 years. The majority were married (88.2%), and nearly half (42.3%) had attained an education level of middle school or higher. Based on the Morse Fall Scale (MFS), 102 participants (21.8%) were classified as low fall risk, 116 (24.8%) as moderate risk, and 250 (53.4%) as high risk.

**Table 1:** Characteristics of the participants (*n* = 468).

Characteristics	*n* (%)
Gender	
Male	262 (56.0)
Female	206 (44.0)
Age, years	
60–69	272 (58.1)
70–79	161 (34.4)
≥80	35 (7.5)
Marital status	
Married	413 (88.2)
Widowed	33 (7.1)
Divorced	22 (4.7)
Place of residence	
Urban area	388 (82.9)
Rural area	80 (17.1)
Monthly income, RMB	
<3,000	419 (89.5)
≥3,000	49 (10.5)
Educational level	
Primary and below	270 (57.7)
Middle school	159 (34.0)
College and above	39 (8.3)
Disease history	
Hypertension	218 (46.6)
Heart disease	86 (18.4)
Diabetes	93 (19.9)
Cerebrovascular disease	68 (14.5)
BMI categories, kg/m^2^	
Low (<18.5)	39 (8.3)
Normal (18.5–23.9)	401 (85.7)
Overweight (≥24.0)	28 (6.0)
Current smoking	113 (24.1)
Current drinking	135 (28.8)
Difficulty in falling asleep	101 (21.6)
Mobility status	
Walking unassisted	414 (88.5)
Walking aid	54 (11.5)
Medical payment	
Medical insurance	317 (67.7)
Self-paying	151 (32.3)
Living status	
Live alone	22 (4.7)
Live with others	446 (95.3)
Hospitalization period, days	
<7	382 (81.6)
7–14	58 (12.4)
>14	28 (6.0)
Operation during hospitalization	145 (31.0)
Fall history	85 (18.2)
MFS score	
0–24	102 (21.8)
25–44	116 (24.8)
≥45	250 (53.4)

BMI, Body mass index; MFS, Morse Fall Scale; Education level: primary and below refers to ≤6 years education; middle school refers to junior and senior secondary education; college and above refers to junior college, bachelor’s degree, and higher.

### 3.2 Latent profiles analysis of fall risk perception

**Figure 2:**
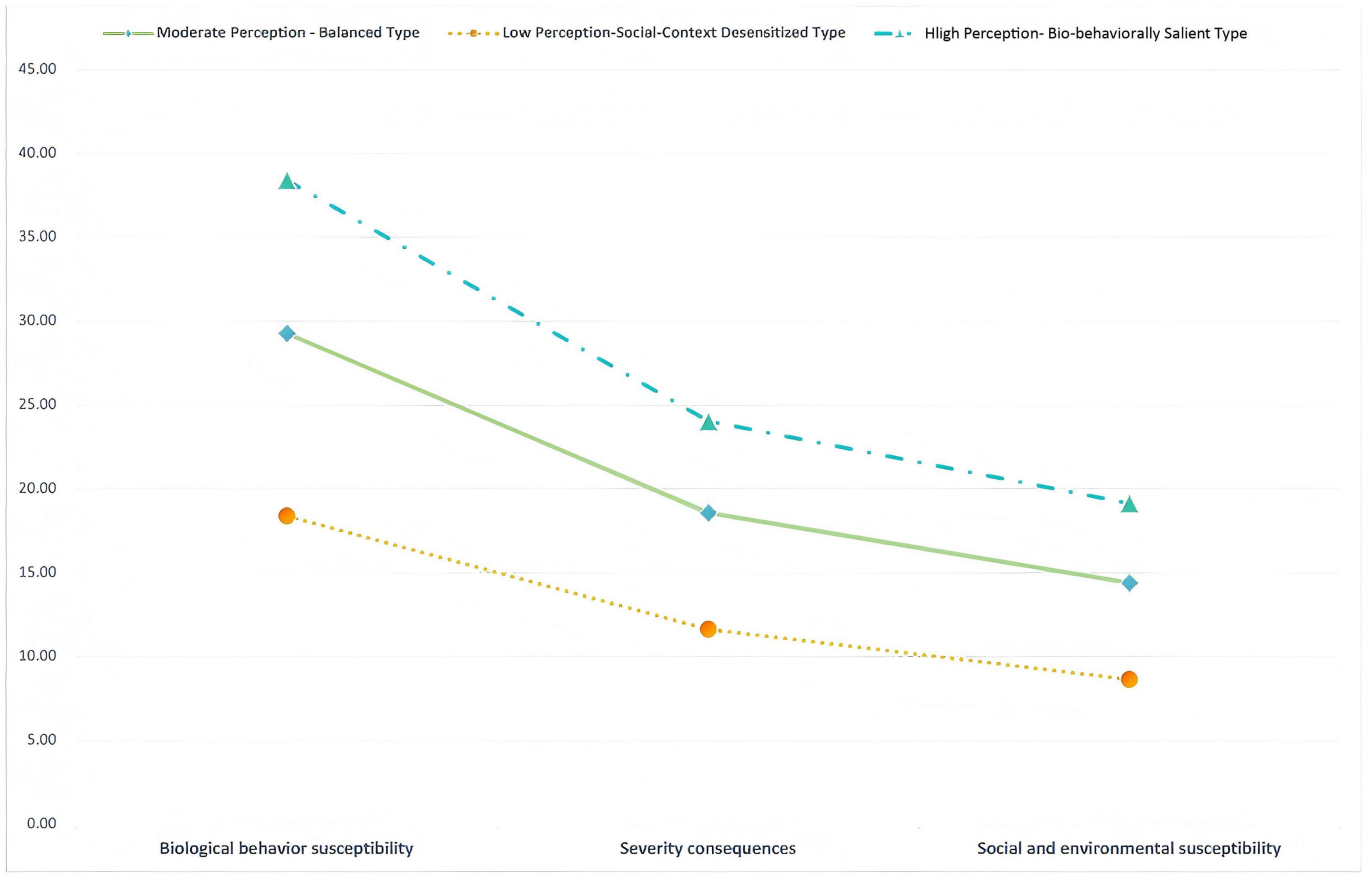
Distribution of three potential classes of fall risk perception.

Table 2 presents the fit indices for latent profile Models 1 through 4. With increasing class number, the information criteria (AIC, BIC, and aBIC) progressively decreased, reaching their lowest values in the four-class model (M4), suggesting improved fit. However, entropy increased from the two-class (0.909) to the three-class model (0.936) but showed no further improvement in the four-class model, indicating that classification precision did not benefit from adding a fourth class. The LMR and BLRT were significant for the three-class model (*p* < 0.001), supporting its superiority over the two-class model. Moreover, all class proportions in the three-class solution exceeded 5%, with relatively balanced distribution. Although M4 exhibited the lowest information criteria, it did not yield higher classification accuracy or interpretability. Therefore, the three-class model was selected as the optimal solution based on statistical fit, parsimony, and theoretical interpretability. Figure 2 illustrates the distribution of participants across the three latent classes.

**Table 2:** Model fit indices of latent profile analysis of presenteeism (*n* = 468).

Model	AIC	BIC	aBIC	Entropy	*p*-value		Latent class probability
					LMR	BLRT	
1 class	8900.09	8924.98	8905.938	/	/	/	/
2 class	7932.196	7973.68	7941.942	0.909	<0.001	<0.001	186 (39.8%) / 282 (60.2%)
3 class	7400.798	7458.877	7414.444	0.936	<0.001	<0.001	203 (43.4%)/137(29.2%)/ 128(27.4%)
4 class	7135.325	7209.998	7152.87	0.936	<0.001	<0.001	102 (21.8%)/120(25.6%)/152 (32.5%)/94 (20.1%)

AIC, Akaike information criterion; BIC, Bayesian information criterion; aBIC, sample size-adjusted BIC; LMR, Lo–Mendell–Rubin Likelihood Ratio Test; BLRT, Bootstrap likelihood ratio test.

### 3.3 Characteristics of classes

Table 3 shows the characteristics of participants in each fall risk perception group. The age groups, education level, difficulty in falling sleep, operation during hospitalization, fall history, and MFS score were significant between three fall risk perception groups.

**Table 3:** Characteristics of individuals among risk perception categories [*n* = 468, *n* (%)].

Variables	Class 1 (*n* = 203)	Class 2 (*n* = 137)	Class 3 (*n* = 128)	*χ*^2^/H	*p*
Gender					
Male	111 (54.7)	76 (55.5)	75 (58.6)	0.508	0.776
Female	92 (45.3)	61 (44.5)	53 (41.4)		
Age, years					
60–69	129 (63.5)	83 (60.6)	60 (46.9)	16.152	**0.003**
70–79	54 (26.6)	47 (34.3)	60 (46.9)		
≥80	20 (9.9)	7 (5.1)	8 (6.2)		
Marital status					
Married	183 (90.1)	119 (86.9)	111 (86.7)	3.842	0.428
Widowed	12 (5.9)	13 (9.5)	8 (6.3)		
Divorced	8 (4.0)	5 (3.6)	9 (7.0)		
Residential location					
Urban area	161 (79.3)	116 (84.7)	111 (86.7)	3.466	0.177
Rural area	42 (20.7)	21 (15.3)	17 (13.3)		
Monthly income, RMB					
<3,000	180 (88.7)	124 (90.5)	115 (89.8)	0.314	0.855
≥3,000	23 (11.3)	13 (9.5)	13 (10.2)		
Educational level					
Primary and below	119 (58.6)	89 (65.0)	62 (48.4)	10.226	**0.037**
Middle school	72 (35.5)	36 (26.3)	51 (39.9)		
College and above	12 (5.9)	12 (8.7)	15 (11.7)		
Hypertension	90 (44.3)	65(47.4)	63 (49.2)	0.811	0.667
Heart disease	34 (16.7)	27 (19.7)	25 (19.5)	0.634	0.728
Diabetes	39 (19.2)	27 (19.7)	27 (21.1)	0.178	0.915
Cerebrovascular disease	31 (15.3)	20 (14.6)	17 (13.3)	0.251	0.882
Body mass index, kg/m^2^					
Low (<18.5)	22 (10.8)	5 (3.6)	12 (9.4)	5.850	0.211
Normal (18.5–23.9)	169 (83.3)	123 (89.8)	109 (85.1)		
Overweight (24.0–27.9)	12 (5.9)	9 (6.6)	7 (5.5)		
Current smoking	45 (22.2)	42 (30.7)	26 (20.3)	4.632	0.099
Current Drinking	61 (30.0)	47 (34.3)	27 (21.1)	5.881	0.053
Activity status					
Walking unassisted	182 (89.7)	125 (91.2)	107 (83.6)	4.292	0.117
Walking aid	21 (10.3)	12 (8.8)	21 (16.4)		
Difficulty in falling asleep	55 (27.1)	15 (10.9)	31 (24.2)	13.322	**0.001**
Medical payment					
Medical insurance	147 (72.4)	91 (66.4)	79 (61.7)	4.261	0.119
Self-paying	56 (27.6)	46 (33.6)	49 (38.3)		
Mode of living					
Live alone	8 (3.9)	7 (5.1)	7 (5.5)	0.481	0.786
Live with others	195 (96.1)	130 (94.9)	121 (94.5)		
Hospitalization period, days					
<7	167 (82.3)	109 (79.6)	106 (82.8)	5.624	0.229
7–14	23 (11.3)	16 (11.7)	19 (14.9)		
>14	13 (6.4)	12 (8.7)	3 (2.3)		
Operation during hospitalization	42 (20.7)	45 (32.8)	58 (45.3)	22.572	**<0.001**
Fall history	32 (15.8)	8 (5.8)	45 (35.2)	39.652	**<0.001**
MFS score	41.31 ± 1.30	37.48 ± 1.54	49.65 ± 2.14	14.905	**0.001**
FRS-COA score					
Biological behavior susceptibility dimension, score, mean ± SD	29.32 ± 4.11	18.31 ± 4.03	38.48 ± 2.46	376.929	**<0.001**
Severity consequences dimension, score, mean ± SD	18.60 ± 2.03	11.61 ± 2.68	24.07 ± 1.35	389.734	**<0.001**
Social and environmental susceptibility dimension, score, mean ± SD	14.39 ± 1.95	8.64 ± 1.69	19.18 ± 1.26	393.073	**<0.001**

Class 1: Moderate Perception - Balanced Type; Class 2: Low Perception-Social Context Desensitized Type; Class 3: High Perception–Bio-behaviorally Salient Type; MFS: Morse Fall Scale; FRS-COA: The Fall Risk Perception Scale for Community-dwelling Older Adults; SD: Standard deviation. Bold values indicate *p* < 0.05.

Class 1, “Moderate Perception - Balanced Type” comprised 43.4% (203/468) of the sample. This group showed moderate and balanced levels across all dimensions of fall risk perception, which reflects their stable and well-rounded awareness toward fall risk.

Class 2, “Low Perception-Social Context Desensitized Type,” comprised 29.2% (137/468) of the sample. This class had the lowest scores, especially in social and environmental susceptibility (8.64 ± 1.69), suggesting a general underestimation of fall risk, particularly lacking sensitivity to contextual cues.

Class 3, “High Perception - Bio-behaviorally Salient Type” comprised 27.4% (128/468) of the sample. This class showed the highest levels of perceived biological and behavioral susceptibility (38.48 ± 2.46) than other categories, along with a strong sense of the severity of fall consequences. Their risk perception was primarily centered on intrinsic and behavioral factors and heightened fall risk awareness.

### 3.4 Multinomial logistics regression

Table 4 presents the results of the multinomial logistic regression analysis, using the High Perception – Bio-behaviorally Salient Type as the reference group, *OR*<1 indicate a lower likelihood of being classified into the Moderate or Low perception profiles, and therefore a higher likelihood of belonging to the High Perception profile. Specifically, older adults aged 70–79 (OR = 0.46, 95% CI: 0.27–0.77), those with college education or above (OR = 0.31, 95% CI: 0.13–0.76), those who underwent surgery during hospitalization (OR = 0.26, 95% CI: 0.15–0.43), and those with a history of falls (OR = 0.44, 95% CI: 0.24–0.81) were significantly less likely to be classified into the Moderate Perception – Balanced Type group.

**Table 4:** The multinational logistic regression results.

Variables	Class 1 (*vs* class 3)					Class 2 (*vs* class 3)				
	*B*	SE	OR	95%CI	*p*	*B*	SE	OR	95%CI	*p*
Age, years (ref: 60–69)										
70–79	−0.79	0.27	0.46	0.27–0.77	**0.003**	−0.54	0.29	0.58	0.33–1.02	0.059
≥80	0.87	0.49	2.38	0.91–6.24	0.078	0.22	0.59	1.24	0.39–3.98	0.714
Educational level (ref: Primary and below)										
Middle school	−0.56	0.27	0.57	0.34–0.96	0.035	−0.97	0.30	0.38	0.21–0.69	**0.001**
college and above	−1.17	0.46	0.31	0.13–0.76	**0.011**	−0.87	0.47	0.42	0.17–1.04	0.062
Difficulty in falling asleep (ref: No)										
Yes	0.20	0.29	1.22	0.70–2.15	0.485	−0.91	0.37	0.40	0.20–0.82	**0.013**
Operation during hospitalization (ref: No)										
Yes	−1.37	0.27	0.26	0.15–0.43	**<0.001**	−0.67	0.28	0.51	0.30–0.88	**0.016**
Fall history (ref: No)										
Yes	−0.83	0.31	0.44	0.24–0.81	**0.008**	−1.95	0.44	0.14	0.06–0.34	**<0.001**
MFS score	−0.02	0.01	0.98	0.97–1.00	**0.006**	−0.02	0.01	0.98	0.96–1.00	**0.008**

SE: standard error, OR: odds ratio, 95% CI: 95% confidence interval; Class 1: Moderate Perception–Balanced Type; Class 2: Low Perception-Social Context Desensitized Type; Class 3: High Perception–Bio-behaviorally Salient Type. Bold values indicate *p* < 0.05.

Similarly, individuals with middle school education (OR = 0.38, 95% CI: 0.21–0.69), those who reported difficulty falling asleep (OR = 0.40, 95% CI: 0.20–0.82), those who had undergone surgery during hospitalization (OR = 0.51, 95% CI: 0.30–0.88), and those with a fall history (OR = 0.14, 95% CI: 0.06–0.34) were less likely to belong to the Low Perception – Social Context Desensitized Type group.

Furthermore, higher MFS scores were associated with reduced odds of being classified into either the moderate perception group (OR = 0.98, 95% CI: 0.97–1.00) or the low perception group (OR = 0.98, 95% CI: 0.97–1.00), indicating that individuals with objectively higher fall risk were more likely to perceive themselves as being at higher risk.

### 3.5 Perceived vs. objective fall risk

Compared to the objectively calculated fall risk based on the MFS, 147 participants (31.4%) accurately perceived their fall risk, while 23.1% overestimated it and 45.5% underestimated it (Table 5). Notably, more than half (74%) of individuals classified as high risk underestimated their fall risk.

**Table 5:** Meshing table between perceived fall risk and objective fall risk.

Perceived fall risk categories	MFS fall risk level			Total
	Low	Moderate	High	
Low perception–social context desensitized type	32	28	77	137 (29.2)
Moderate perception–balanced type	45	50	108	203 (43.4)
High perception–bio-behaviorally salient type	25	38	65	128 (27.4)
Total	102 (21.8)	116 (24.8)	250 (53.4)	468 (100)

## 4 Discussion

To our knowledge, few studies have applied a person-centered approach such as LPA to characterize heterogeneity in fall risk perception among older adults returning to community living after discharge. By moving beyond a uniform approach, we identified three distinct and interpretable profiles—Moderate Perception – Balanced Type, Low Perception – Social Context Desensitized Type, and High Perception – Bio-behaviorally Salient Type. These profiles reflect heterogeneous cognitive and contextual patterns of risk perception and offer new insights into how older adults interpret fall threats. By integrating subjective perception with objective risk (MFS scores), we revealed notable mismatches between perceived and actual risk, underscoring the need for tailored fall prevention strategies. Importantly, this profile-based framework complements prior variable-centered work by making perceptual “blind spots” visible at the subgroup level, which is directly relevant for discharge follow-up and community care.

The Moderate Perception – Balanced Type, comprising the largest proportion of participants, reflected a relatively integrated awareness of both intrinsic and extrinsic fall risk factors. In contrast, the Low Perception – Social Context Desensitized Type exhibited markedly reduced sensitivity to environmental and social cues. Compared with the High Perception – Bio-behaviorally Salient Type, both groups were characterized by younger age, lower educational attainment, no prior fall, and no surgical experience—factors that may reflect lower health salience and fewer risk-related experiences. A plausible explanation is that fewer salient “risk cues” may reduce the perceived immediacy of falling, thereby dampening perceived susceptibility and the motivation to attend to environmental hazards, consistent with evidence that adverse health events often heighten perceived vulnerability and prompt protective responses (36). From the perspective of the Health Belief Model, fewer salient risk cues may weaken perceived susceptibility and reduce the activation of cues to action. Lower educational attainment may further constrain risk information processing and awareness of contextual hazards, making social/environmental cues less likely to be recognized or internalized, which aligns with prior work reporting mismatches between subjective perception and objectively assessed fall risk and highlighting cognitive/psychological correlates of such discordance (13–15). Additionally, individuals without difficulty falling asleep were more likely to belong to the low-perception group; this may reflect lower health-related worry or somatic vigilance, which could reduce attention to fall-risk cues, although this relationship requires further confirmation (37). Finally, although the high-perception profile may be more responsive to risk information, heightened perceived susceptibility and severe consequences may also generate distress or activity restriction, suggesting that discharge counseling should balance risk awareness with supportive, action-oriented guidance to avoid excessive fear and maladaptive avoidance (38).

Furthermore, although individuals with objectively higher fall risk were statistically more likely to perceive themselves at higher risk (Table 4), a substantial proportion still misjudged their fall susceptibility: 23.1% overestimated and 45.5% underestimated their risk. Notably, 74% of those classified as high risk based on clinical criteria underestimated their fall risk. This significant discrepancy reveals a critical gap in risk awareness among older adults. Similar perceived–objective mismatches have been reported in clinical settings, such as risk underestimation among patients with dementia in a rehabilitation center (37.6%) (39) and among inpatients (27.5%) (14). Compared with these institutional samples, our participants were assessed at discharge and were about to manage fall risk in the community without continuous professional supervision, which may partly explain the higher underestimation rate. This pronounced underestimation among high-risk individuals may reflect combined cognitive, motivational, and sociocultural mechanisms. Older adults may be less attuned to fall-risk cues (e.g., gait instability, polypharmacy, environmental hazards) and may rely on prior “no-fall” experiences when judging current risk. In addition, optimistic bias or denial to protect autonomy and self-esteem (14). In the Chinese sociocultural context, the stigma of frailty and dependence may further discourage acknowledging vulnerability, as admitting high risk can imply weakness, loss of face, or burdening family. Together, these mechanisms are clinically important because they can weaken engagement with preventive behaviors even when objective risk is high (40). Given that risk perception is a modifiable determinant of health-related behavior (41), it is crucial to identify subgroups with discordant perceptions—particularly those who underestimate their risk—for tailored interventions that enhance awareness and promote behavior change. According to the Health Belief Model, such underestimation may weaken perceived susceptibility and reduce readiness to adopt preventive behaviors. From a clinical perspective, these findings support incorporating subjective risk perception assessments into routine care, enabling clinicians to detect not only physical risks but also perceptual blind spots that may hinder effective prevention. Personalized communication approaches—such as motivational interviewing or visual feedback—could help correct misperceptions and improve responsiveness to clinical advice. Evidence suggests that education tailored to patients perceived fall risk can improve awareness, supporting the feasibility of perception-informed communication (16).

A key strength of this study is the integration of a person-centered perception typology with an objective risk indicator (MFS), which enables identification of clinically high-risk individuals with underestimated perception—an actionable target for discharge follow-up and community-based prevention. In practice, the three profiles can support streamlined, home-focused guidance after discharge: the Bio-behaviorally Salient Type may benefit from reinforcing safe routines around individual triggers (e.g., nighttime toileting, dizziness, footwear, hurried activities); the Moderate Balanced Type may benefit from reinforcing core messages across personal, consequence, and environmental domains; and the Social Context Desensitized Type may benefit from strengthening recognition of home/environmental hazards and aligning perceived risk with objective risk. Future research may build on this by testing whether perception-informed communication improves alignment between perceived and objective risk and increases engagement in fall-prevention self-management after returning home.

This study has several limitations. First, the sample was drawn from a single tertiary hospital in Hangzhou, which may limit the generalizability of the findings to older adults in other geographic regions. Second, our participants were older adults transitioning from hospital to community living, and perceptions measured at discharge may not fully represent those of stable, general community-dwelling older adults. The hospitalization experience (e.g., medical care exposure, fall-related education, and acute health events) may have temporarily heightened fall-risk awareness, potentially leading to higher perceived risk than would be observed in a typical community sample. In addition, although the FRS-COA showed excellent internal consistency in our sample (*α* = 0.964), it was developed for community settings and has not been formally validated for the post-discharge transition; future studies should examine construct validity and measurement invariance across settings. Third, the cross-sectional design limits the ability to draw causal inferences between fall risk perception and associated factors. Longitudinal studies are needed to examine how perceptions evolve over time and how they influence fall-related outcomes. Finally, key psychosocial and cultural variables—such as emotional state, risk preference, and health beliefs—were not included in the analysis, despite their potential influence on individuals’ risk perception. These factors warrant further investigation in future research.

## 5 Conclusion

This study, using a latent profile analysis, identified three distinct fall risk perception profiles among older adults transitioning from hospital to community living. Lower fall-risk perception was more common among relatively younger and less-educated older adults, particularly those without a prior fall history or surgery during hospitalization. Notably, only one-third of participants accurately perceived their fall risk, and underestimation was common even among those objectively at high risk, highlighting the need to identify older adults with discordant or underestimated perceptions at discharge. These profiles can inform streamlined, home-focused discharge guidance: for the high-perception, bio-behavior–salient subtype, prioritize managing individual triggers and converting awareness into safe daily routines; for the moderate balanced subtype, reinforce key messages across personal, consequence, and environmental domains; and for the low-perception subtype, strengthen recognition of home/environmental hazards and align perceived risk with objective risk. Overall, profile-informed communication may support more targeted home-based fall prevention and self-management after discharge.

## Data Availability

The raw data supporting the conclusions of this article will be made available by the authors, without undue reservation.
