# Supplementary material for: Identifying subtypes and determinants of fall risk perception in older adults: a latent profile analysis
Source: Front Public Health. 2026 Mar 26;14:1759157. doi: 10.3389/fpubh.2026.1759157 (PMC13061864; doi:10.3389/fpubh.2026.1759157)
Supplement: Supplementary file 1 [file Table_1.DOCX]

**General Information Questionnaire**

1. **Gender**

A Male B Female

1. **Age(years)**:______
2. **Marital status**

A Married B Widowed C Divorced

1. **Residential location**

A Urban area B Rural area

1. **Monthly income(RMB)**

A <3000 B ≥3000

1. **Educational level**

A Primary and below B Middle school C College and above

1. **Disease history**

A Hypertension B Heart disease C Diabetes D Cerebrovascular disease

1. **BMI categories(kg/m^2^)**

A Low (＜18.5) B Normal (18.5-23.9) C Overweight (≥24.0)

1. **Current smoking**

A Yes B No

1. **Current Drinking**

A Yes B No

1. **Difficulty in falling asleep**

A Yes B No

1. **Activity status**

A Walking unassisted B Walking aid

1. **Medical payment**

A Medical insurance B Self-paying

1. **Mode of living**

A Live alone B Live with others

1. **Hospitalization period(days)**

A ＜7 B 7-14 C ＞14

1. **Operation during hospitalization**

A Yes B No

1. **Fall history**

A Yes B No
